# The Development of Eletriptan Hydrobromide Immediate Release Buccal Films Using Central Composite Rotatable Design: An In Vivo and In Vitro Approach

**DOI:** 10.3390/polym14193981

**Published:** 2022-09-23

**Authors:** Waqar Siddique, Muhammad Zaman, Rai Muhammad Sarfraz, Muhammad Hammad Butt, Atta Ur Rehman, Noman Fassih, Ghadeer M. Albadrani, Roula Bayram, Mohammad Y. Alfaifi, Mohamed M. Abdel-Daim

**Affiliations:** 1 College of Pharmacy, University of Sargodha, Sargodha 40100, Pakistan; 2 Department of Pharmacy, University of South Asia, Lahore 54000, Pakistan; 3 Faculty of Pharmacy, University of Central Punjab, Lahore 54000, Pakistan; 4 Department of Medicinal Chemistry, Faculty of Pharmacy, Uppsala University, 75123 Uppsala, Sweden; 5 Department of Pharmacy, Forman Christian College, Lahore 54000, Pakistan; 6 Department of Medical Cell Biology, Faculty of Medicine, Uppsala University, 75123 Uppsala, Sweden; 7 Department of Biology, College of Science, Princess Nourah Bint Abdulrahman University, P.O. Box 84428, Riyadh 11671, Saudi Arabia; 8 Pharmacy Program, Department of Pharmaceutical Sciences, Batterjee Medical College, Jeddah 21442, Saudi Arabia; 9 Biology Department, Faculty of Science, King Khalid University, Abha 9004, Saudi Arabia; 10 Pharmacology Department, Faculty of Veterinary Medicine, Suez Canal University, Ismailia 41522, Egypt

**Keywords:** anti-migraine, buccal films, rapid onset, oral cavity, characterization

## Abstract

The objective is to develop immediate release buccal films of Eletriptan Hydrobromide (EHBR) using hydroxypropyl methylcellulose (HPMC) E5. The buccal films have the ability to disintegrate rapidly and provide both systemic and local effects. The solvent casting method was employed to prepare the films and the central composite rotatable design (CCRD) model was used for film optimization. All the formulated films were characterized for physicochemical evaluation (Fourier transform infrared spectroscopy (FTIR), X-ray Diffraction (XRD), differential scanning calorimetry (DSC), and Scanning electron microscopy (SEM), in in-vitro, ex-vivo, and in-vivo drug release. The fabricated films were transparent, colorless, and evenly distributed. The FTIR spectra showed no chemical interaction between the drug and excipients. In in-vitro analysis, the film has the highest% drug release (102.61 ± 1.13), while a maximum of 92.87 ± 0.87% drug was diffused across the cellulose membrane having a pore size of 0.45 µm. In the ex-vivo study, drug diffusion across the goat mucosa was performed and 80.9% of the drug was released in 30 min. In-vivo results depict a mean half-life (t½) of 4.54 ± 0.18 h and a C_max_ of 128 ± 0.87 (ng/mL); T_max_ was achieved in 1 h. Furthermore, instability and histopathological studies buccal films were proven to be safe and act as an effective dosage form. In a nutshell, optimized and safe instant release EHBR buccal films were prepared that have the tendency to provide effect effectively.

## 1. Introduction

The oral drug administration route is the oldest and most suitable route of drug administration [1]. The oral cavity is highly enriched with blood capillaries and is more permeable to low molecular drugs than any other route [2]. This route can be used for both systemic and localized effects. However, this route encounters limitations, such as swallowing distress, especially in conditions, such as vomiting, dysphagia, and Parkinson’s disease. At least 28% of the overall population showed uneasiness consuming the solid dosage form [1]. In the 1970s, a new dosage form was introduced to overcome these disadvantages and was termed fast dissolving films (FDF) [3]. These films are prepared using hydrophilic polymers that become hydrated and stick in the oral cavity upon encountering salivary fluids. After hydration, these polymers disintegrate the drugs in the oral cavity due to a network of capillaries in sublingual mucosa, causing an increase in systemic absorption of the drug [4]. These films are soft and flexible, due to which they remain in the oral cavity. Additionally, they take a longer time to disintegrate, causing an increased residence in the oral cavity, and ultimately reducing the dosing frequency [5].

The main constituents of these films are polymers that may be natural, synthetic, or semi-synthetic. Disintegration time (DT) depends on the polymer’s nature, which affects the drug release rate, and mechanical properties [6]. A promising function of these films is mucoadhesion, a process by which the polymers, despite their origin, adhere to the oral cavity. The mechanism of mucoadhesion provides for the phenomenon of wetting and then entanglement of polymer chains at the mucous side within the oral cavity. The most commonly used polymer is hydroxypropyl methylcellulose (HPMC) [7]. As the polymer becomes hydrated in oral mucosa, it develops gel-like consistency. Other additives, such as plasticizers and surfactants, are also added to formulations to confer desirable properties [8].

Low molecular weight and non-volatile plasticizers are used to improve the flexibility of the films. Plasticizers are chosen based on polymer and film compatibility. The mechanical strength of the films is dependent upon the concentration and type of plasticizer used. Natural polymers are currently used more widely, including glycerol, polyethylene glycol, and propylene glycol [9]. Incorporation of surfactant within formulation improves the DT of films and thus improves drug release. Both surfactant and plasticizer enhance the properties of formulated films [4].

Approximately 1 billion people are affected by migraines worldwide [10]. Women (20.7%) are affected more than men (9%). Migraine is characterized by harsh throbbing pain which lasts from 4 to 72 h [11,12]. Further, it is marked by some symptoms, including gastric distress and photophobia [13,14,15]. The condition worsens when the patient cannot take medication due to a high frequency of pain and nausea [15].

Triptans are the first line of therapy for migraineurs. They can activate 5-HT_1B_ and 5-HT_1D_ receptors and cause vasoconstriction [16]. Eletriptan hydrobromide (EHBR) is considered a safe and effective medication for severe to moderate migraine attacks [17]. The drug achieves 50% bioavailability after oral administration [18,19]. Its peak plasma concentrations (T_max_) are achieved within 1 h [20,21]. EHBR is approximately 85% protein-bound [22]. While, the high first-pass effect demands another route of drug administration [17,23].

The current study aimed to develop and characterize an optimized buccal film by incorporating EHBR to ensure a safe and prompt effect. A total of 13 runs were made by using Design Expert, followed by a central composite rotatable design (CCRD). Two variables were chosen, including HPMC E5 as X_1_ and glycerol as X_2_ variables. Different responses, including disintegration time (DT), total dissolving time (TDT), folding endurance (FE), and drug release were studied.

## 2. Materials and Methods

EHBR was received as a gift by Wilshire labs, Lahore, Pakistan. Highnoon Laboratories, Lahore, Pakistan gifted HPMC E5 (Molar mass: 324.285), Tween 80, and glycerol. Ethanol and all other chemicals of analytical grade were purchased from Fisher Chemical, Loughborough, UK.

### 2.1. Preparation of Film Formulations

Films were prepared by using the solvent evaporation method [24]. To make the procedure more convenient, solutions of the drug and each of the excipients were prepared separately. Firstly, the drug solution was prepared using ethanol as solvent under continuous stirring on a magnetic stirrer for 5 min at 500 rpm. Afterward, 4% aqueous solution of HPMC E5, 5% of Tween 80, and 3% of glycerol were prepared in separate beakers and used according to the formulation design (Table 1 and Table 2). Initially, the polymeric solution was subjected to stirring using a magnetic stirrer, followed by the addition solution of Tween 80, glycerol, and the drug, one by one until a homogenous mixture was formed [25,26,27]. The solution was covered by aluminum foil to prevent evaporation. The final mixture was then poured into Petri dishes (Norman, China) and allowed to dry at 40 °C for 24 h in the oven. After that, the prepared films were peeled off and kept in aluminium foil for further use [26].

### 2.2. Analytical Method

Regarding better elution properties of EHBR-based oral solution and film formulation through high-performance liquid chromatography (HPLC), the wavelength was adjusted to 220 nm. High-performance liquid chromatographic analysis was conducted to study the pharmacokinetics of the drug, both in film formulation as well as in oral solution (Reference formulation). For this purpose, 1260 Infinity II LC System, Agilent Technologies, Santa Clara, CA, USA, equipped with a C 18 column having dimensions of 250 mm × 4.6 mm and a particle size of 5 μm was used. Different combinations of the solvent systems were tried to find better elution of the drug, however, the mobile phase consisted of phosphate buffer and acetonitrile in a 70:30 ratio, with adjusted pH to 4.6, a column flow of 1 mL/min at ambient temperature, and a detection wavelength of up to 220 nm [28].

### 2.3. Numerical Optimization

A widely used software to optimize formulations is Design Expert, which was used to optimize the relation between different variables by varying their concentrations [29]. In the current studies, CCRD was used to foresee 13 runs reliant upon the variables. The data was subsequently fitted in the polynomial equation (Equation (1)), and analysis of variance (ANOVA) with a 95% confidence interval was employed [30]:

Y = β_0_ + b1X_1_ + b_2_X_2_ + b_12_X_1_X_2_ + b_1_X_12_ + b_2_X_22_ (1)

β_0_ represents the arithmetic mean response of the selected runs, and Y represents the dependent variable. β_1_ is the estimated coefficient for the factor X_1,_ X_2_. In this study, we used two variables designated X_1_ and X_2_, which were changed from low to high values. A polynomial equation judges the positive or negative result [31].

### 2.4. Characterization of Films

All the prepared instant release buccal films were characterized for their organoleptic, physical, and chemical evaluation.

#### 2.4.1. Organoleptic Evaluation

Prepared films were evaluated for the presence of any lumps, bubble formation, and any color deposition [32].

#### 2.4.2. Weight Variation

The prepared films were cut into 2 × 2 cm^2^ dimensions, followed by weighing of individual film strips, using a digital weighing balance. All measurements were performed in triplicate, and the average weight was calculated [32].

#### 2.4.3. Film Thickness

The formulated films were subjected to a thickness test, using a digital micrometer (Mitutoyo, IP65, Waltham, MA, USA). Thickness from three different locations from each strip was determined and their average thickness was calculated [33].

#### 2.4.4. Content Uniformity

Uniformity of content is an important test to ensure homogeneity in the distribution of the drug and polymer within film preparation. Three strips each of 1 cm^2^ in size were cut and dissolved in 5.7 pH buffer under continuous stirring, using a magnetic stirrer. The solution was filtered using syringe filters (0.22-μm, Sterlitech, Bedford, MA, USA) of nylon material to remove undissolved particles. The prepared mixture was analyzed using a UV-Visible double beam spectrophotometer (Halo DB-20 UV, Progen Scientific, Livingston, UK) at 220 nm wavelength [34]. The content uniformity of each formulation was determined in triplicate and finally calculated by using the following Equation (2).

(2) $$\%\;\mathrm{drug}\;\mathrm{content}=\frac{\mathrm{Actual}\;\mathrm{amount}}{\mathrm{Theoretical}\;\mathrm{amount}}\times 100$$

#### 2.4.5. Surface pH

The pH of the developed films is an important parameter. As films are placed in the buccal cavity, so, to avoid any damage to the cavity, the pH of the formulation must be in accordance with the buccal mucosa. Strips of films were cut into 1 cm^2^ and the surface was moistened by using a few drops of distilled water. The pH of wetted films was analyzed using a pH meter (Bench Meter, AD1030 Professional, Adwa, Hungary). The bulb of the pH electrode was touched to the films and, after stabilizing, the pH value was determined in triplicate [34].

#### 2.4.6. FTIR Analysis

FTIR analysis was done to determine any interaction between the used active ingredient and the excipients. Bruker Alpha II (Compact series, Fremont, CA, USA) apparatus was used within the range of 500 to 4000 cm^−1^ wavelength [35].

#### 2.4.7. DSC Analysis

Differential Scanning Calorimeter (Q2000, TA Instruments, New Castle, DE, USA) was used to perform the DSC analysis. This test was performed on formulated film and pure drugs. The sample size of 8 mg for both EHBR and EHBR-based film was carefully weighed and encapsulated in pans of aluminum metal. The instrument was earlier calibrated by using indium and zinc. For the sake of analysis, the Nitrogen gas at a flow rate of 50 mL/minute was used. The heating of the sample was done between 40 to 280 °C and maintained at 20 °C/min [36,37].

#### 2.4.8. X-Ray diffraction (XRD)

XRD of pure drug sample and formulated film of EHBR was determined using X-Rays Diffractometer (JDX-3532 JEOL, Tokyo, Japan) equipped with CuKa (Wavelength = 1.5418 Å). The current was set at 10 mA and the voltage at 40 kV. Scanned at 2°/min and 2 theta ranges from 0 to 160°. The 2 theta values, and intensities of the samples were scanned, and the graph was plotted. XRD ascertained whether the sample was crystalline or amorphous [38].

#### 2.4.9. Optical Microscopy

Prepared films were studied at a micro-level using an optical microscope at 40× magnification [39].

#### 2.4.10. Surface Electron Microscopy (SEM)

The optimized formulation of EHBR was evaluated using FEI Nova 450 Nano SEM (Thermo Fisher, Waltham, MA, USA) under different resolutions ranging from 1000× to 10,000× [40].

#### 2.4.11. Percentage Moisture Content

Karl Fischer titration (KFT) was used to determine the moisture content [41] present in formulated films at 80 °C.

#### 2.4.12. Disintegration Time (DT) and Total Dissolving Time (TDT)

Formulated films were placed in a 10 mL saliva-based buffer having 5.7 pH, which was maintained at 37 °C and agitated slowly. The time in which films start to crack is termed DT, while the time in which the film completely dissolves in buffer was termed TDT [42,43].

#### 2.4.13. Mechanical Strength

The mechanical strength, including tensile strength (TS) and folding endurance (FE), is the most promising factor in determining the integrity of the film preparation. TS and FE ensure that the films endure the applied stress or pressure through manufacturing, packaging, and transportation [44].

#### 2.4.14. Tensile Strength (TS)

The mechanical strength of the developed films was determined using a Tensile strength tester (Lloyd Instruments, LS series, Ametek, British Columbia, Vancouver, Canada). The film with an area of 5.7 × 1 cm^2^ was placed between the two jaws of the machine. The lower jaw moves downward, while the upper jaw is fixed in position. The point at which the film was ruptured was determined by using Equation (3).

TS = F/A
(3)

where F represents the breaking load measured in units of Newton’s (N), and the area was designated as A measured in units of centimeter square (cm^2^) [45].

#### 2.4.15. Percentage Elongation at break (% EB)

% EB is determined using the film’s original length, thickness, and width until it breaks under the influence of applied force [46]. It was calculated using Equation (4).

%EB = L2/L1 × 100
(4)

where L1 is the film’s original length and L2 is the change in length at the break.

#### 2.4.16. Strain

When a force is applied to film, it causes an extension in film, referred to as a strain. The strain is used to directly measure the film’s elastic behavior calculated by using Equation (5) [47].

Strain = ((L2 − L1))/(L1)
(5)

The film’s original length was represented as L1, while the change in length was presented as L2.

#### 2.4.17. Folding Endurance (FE)

The FE of formulated films determines the film’s strength and integrity. Strips from formulated films were folded at 180° at the same point until the films were ruptured. The number of times after which the film was ruptured was determined. The test was done in triplicate, and the mean was calculated [48].

#### 2.4.18. In Vitro Drug Release Studies

The dissolution paddle apparatus was used to determine in vitro drug release studies. A simulated saliva-based buffer (used as dissolution media) having 5.7 pH was used for dissolution purposes. Film strips having a dimension of 2 × 2 cm^2^ (equivalent to a one-time dose of EHBR, which is 20 mg) were cut and fastened on a glass slide. The glass slide was dipped into a dissolution basket. Baskets were already filled with 250 mL of dissolution media. The media temperature was set at 37 ± 0.5 °C and paddles were rotated at 50 rpm. Samples were withdrawn after 2, 4, 6, 8, 10, 15, 20, 25, 30 min. A sample size of 5 mL was withdrawn each time, which was replaced with the same amount of buffer maintained at the same temperature (mentioned above 37 ± 0.5 °C). At the end of the test, the absorbance of the samples was analyzed at 220 nm using a UV-Vis spectrophotometer [25].

#### 2.4.19. In Vitro Diffusion Studies

Franz diffusion cell was used to determine the drug diffusion studies. The cell has a receptor compartment of 5 mL, which was filled with a simulated saliva-based buffer having pH 5.7, and its temperature was maintained at 37 ± 0.5 °C. The formulated films were placed on cellulose acetate membranes, having a pore size of 0.45 µm. The prepared films were placed on the membrane. The placement of films was such that the drug diffusion faces toward the receptor chamber. Magnet speed was set at 50 rpm. The sample size of 1 mL was withdrawn, while the remaining conditions remained the same, as mentioned above [49].

#### 2.4.20. Kinetic Modeling

The following statistical methods were applied to determine drug release patterns from formulations, including ANOVA, Model dependent methods (first order, zero order, Hixson Crowell model, Korsmeyer Peppas model, and Higuchi model), and model-independent models (difference factor, similarity factor).

#### 2.4.21. Numerical Optimization

The formulations were optimized based on DT, TDT, in vitro drug release, and drug diffusion by using the Design Expert software. The results were interpreted by using the software, and estimated results were designed using polymer, plasticizer, and Tween 80 in 3.87 mL, 0.7 mL, and 0.155 mL concentrations.

#### 2.4.22. Ex Vivo Permeation

After numerical optimization, an optimized film was selected and further tests, which included ex vivo and in vivo analysis, were performed. Fresh excised goat (a local breed of Sahiwal) buccal mucosa was used in 2 h, as received from the slaughterhouse. For 2 h, the mucosa was placed in a normal saline solution. Underlying tissues and fat of mucosa were properly cleaned. It was then placed in Franz’s cell; meanwhile, all conditions remained the same, as discussed [50].

#### 2.4.23. In Vivo Analysis

As there appeared no commercial EHBR film in the market, EHBR films were compared with the EHBR dispersion.

#### 2.4.24. Selection of animals

For in vivo analysis, albino rabbits with an average weight of 3 kg were selected. Animals were provided with an ad libitum quantity of water, but they were kept fastened for a period of approximately 24 h before the start of analysis [51]. The study was conducted according to the guidelines provided by the ethical committee of the University of Central Punjab (Ref. No.: UCP/IRC/2019/144) to conduct animal studies. All male rabbits, having good health, an average weight of at least 3 kg, and no previous history were included in the study. However, underweight rabbits those showing any signs of disease, or having a history of medications were excluded.

#### 2.4.25. Dose Administration

Rabbits were given an oral drug dispersion and buccal film (for comparison purposes) according to their body weight which was 1 mg/kg. Meanwhile, during the administration of buccal films, the animals were anesthetized lightly [52] by using diazepam intramuscular (IM) injection at a dose of 1 mg/kg of their body weight in conjunction with 10 mg/kg of ketamine. As soon as the animals were anesthetized, butterfly catheters were instilled in their marginal ear vein [53]. Prepared films were then placed and gently pressed on the buccal mucosa of the animal for adhesion. Then, 1 mL of blood was removed from the marginal ear vein of the rabbit at a time interval of 0, 1, 2, 3, 4, 6, 8, 12, and 24 h and rapidly transferred to heparinized tubes [54,55].

#### 2.4.26. Drugs Extraction from Animal Plasma

The collected samples were centrifuged at 5000 rpm for 10 min. The supernatant was separated and vortex again for 10 min with the addition of 3 mL methanol and centrifuged again at 5000 rpm for 20 min. The supernatant was again transferred in a test tube containing 3 mL of diethyl ether and centrifuged at 5000 rpm for 10 min. The organic solvent was dried at 40 °C by placing the test tubes in the water bath. Obtained samples were then reconstituted with a mobile phase of approximately 200 μL out of which 20 μL was further used for the HPLC study [55]. Samples were analyzed at 220 nm wavelength. Different pharmacokinetic factors were studied, including T_max_, C_max_, t½, area under the curve (AUC) at the first moment (AUC 0-t) (ng/mL × h), and mean residence time (MRT) (h).

#### 2.4.27. Statistical Analysis

One-way ANOVA at a confidence interval of 95% was performed on results obtained from experimental work. Differences were statistically significant if *p* < 0.05 [56]. Trials were conducted at n = 6, and their mean ± SD was recorded.

#### 2.4.28. Stability Studies

Formulated films were placed in a stability chamber to identify their stability. Films were placed at 40 °C and 75% ± 5% relative humidity (RH) [57,58] and 30 °C and 65% ± 5% RH [30,59] for a period of 3 months [57]. At the end of 3 months, films were investigated for drug content, appearance, in vitro drug release study [60], TDT, DT, and FE.

#### 2.4.29. Histopathological Slides

At the end of the experiment, rabbits were sacrificed, and their major body organs which include buccal mucosa, heart, liver, kidneys, and lungs, were removed. The organs were placed in a 10% formalin solution and further studied for the formation of any lesions or signs of abnormality [61,62,63].

## 3. Results and Discussions

### 3.1. Organoleptic Evaluation

It was observed that formulated films were transparent, had no odor, and were flexible with no bubble formation.

### 3.2. Weight Variation

Weight variation of formulated films ranges from 0.013 ± 0.0004 for F12 to 0.027 ± 0.0004 mg for F4. It is an important parameter to demonstrate the even distribution of polymer, drug, and excipients within the formulation, as uneven distribution of ingredients may cause dose dumping, as seen in a previous study conducted by Kassem et al. in which uniform and even weight films were formulated by using the solvent casting method [64].

### 3.3. Film Thickness

Film thickness varied from a maximum of 0.191 ± 0.023 mm for F8 and a minimum of 0.091 ± 0.023 mm for F2. Results showed that all formulated films were of uniform thickness and could be easily used for buccal administration [27].

### 3.4. Content Uniformity

The content uniformity fell between 97.16 ± 2.5% to a maximum of 104.6 ± 1.7% for F1 and F10, respectively. As per United States Pharmacopeia (USP) guidance, drug contents could be varied between 90% to 110%. The results suggested the drug was evenly distributed throughout the film [65].

### 3.5. Surface pH

The pH of the oral cavity ranges from 5.5 to 7 [66,67]. The pH of all the formulations ranged between 5.85 ± 0.13 for F4 to 6.2 ± 0.13 for F1, respectively. As pH was in accordance with the oral cavity, it is plausible to predict no interaction with the buccal cavity.

### 3.6. FTIR Analysis

FTIR of Tween 80 showed a band at 1717 cm^−1^, possibly due to C=O and reinforced the presence of α, β-unsaturated ester. In comparison, a sharp band at 1121 cm^−1^ confirms C-O extending. FTIR spectrum of pure HPMC E5 demonstrated a broad absorption band at 1050 cm^−1^ possibly due to the CO-O-CO group. FTIR band of glycerol indicated an extending at 3288, which confirmed the presence of the carboxylic group. In contrast, alkanes were established at the peak of 2878 cm^−1^.

**Figure 1:**
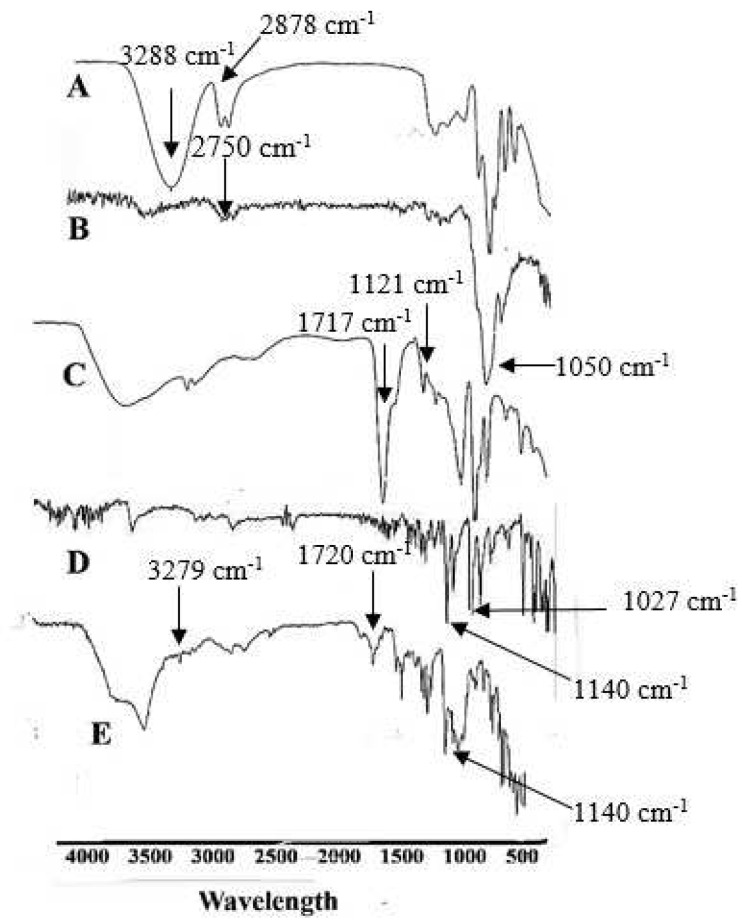
Represents the FTIR spectrum (**A**) Glycerol (**B**) HPMC E5 (**C**) Tween 80 (**D**) EHBR (**E**) Film.

Meanwhile, a sharp stretch confirms the presence of C-O at 1027 cm^−1^ [68]. FTIR of EHBR represents a sharp peak at 1140 cm^−1^, which depicts a strong C-O bond and the presence of aliphatic ether. Finally, the FTIR spectrum of film elaborated that the drug’s peak, previously at 1140 cm^−1^, remained at 1140 cm^−1^. In the case of glycerol, an O-H stretch was observed at 3288 cm^−1^ before the formulation; the same peak was observed at 3279 cm^−1^ after the formulation, suggesting no significant interaction between the drug and excipients, as shown in Figure 1.

### 3.7. Differential Scanning Calorimetry (DSC) Analysis

It was used as an analytical method to determine the thermal properties of drugs and polymers. DSC graphs were plotted by taking heat flow values on the y-axis and temperature on the x-axis. It is widely used for the decomposition performance of materials employed [69].

**Figure 2:**
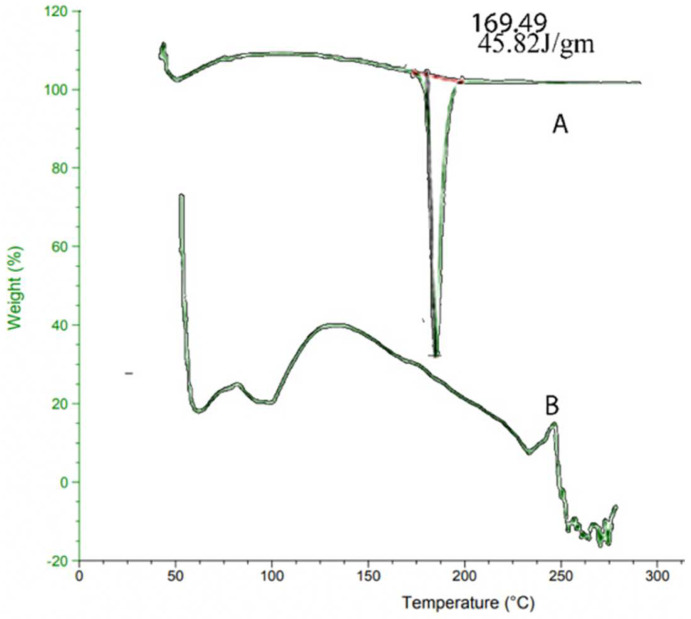
DSC pattern for (A) Pure drug (B) Formulation.

In the case of EHBR, as shown in Figure 2, glass transition temperature (Tg) was observed at ~40 °C, which is supported by an exotherm attributable to crystallization (Tc), having onset at ~169.49 °C and a melting temperature (Tm) at ~173.93 °C. As the concentration of glycerol content increases, a decrease in glass transition, crystallization, and melting temperatures was observed [70]. In the EHBR film, Tg was observed at ~40 °C, supported by an exotherm due to Tc, having onset at ~92 °C and Tm at ~225 °C. DSC of pure drug EHBR showed a sharp temperature peak at 169.49 °C, as represented in Figure 2 [71]. DSC of film formulation changes the endotherms and now appears at 235 °C, suggesting reduced purity of ingredients due to the mixing of drug and excipients that resulted in the broader melting point [72].

### 3.8. X-ray Diffraction (XRD)

**Figure 3:**
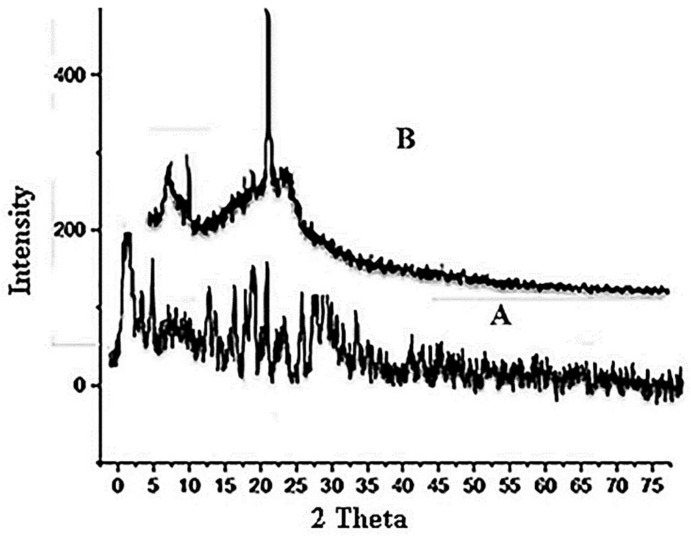
XRD pattern (A) Pure drug (B) Formulation.

The pure drug form appears to be crystalline, and it exhibits visible peaks at 2 θ = 11°, 22°, and 28°, respectively [73]. Following film formulation, XRD of films demonstrated the same peaks at 2 θ = 11°, 22°, suggesting no interactions between the drug and the developed formulation, as shown in Figure 3. However, the peak intensity of pure EHBR drug is reduced in the case of EHBR-based films, which could be possible because the physical mixture decreases the crystalline nature of the drug, and now it shifts toward its amorphous form. After, film formulation XRD of films exhibited a fused peaks pattern, which suggested that the drug is stable in the film formulation as well and merged fully with the used polymer [74].

### 3.9. Optical Microscopy

**Figure 4:**
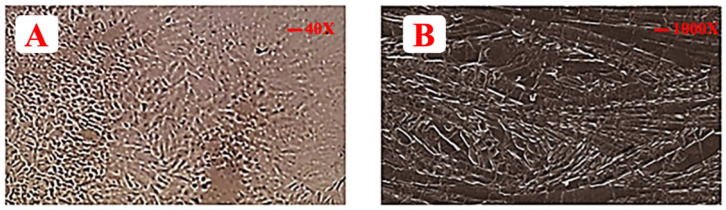
Illustrating (**A**) optical microscope image of EHBR film and (**B**) SEM image of EHBR film.

Optical microscopy was done at 40× magnification. Meanwhile, a continuous texture showed a complete mesh of polymer in which drug and excipients are dispersed completely, as evident from Figure 4.

### 3.10. SEM

The SEM of formulated films revealed some larger broken pieces that were formed during the process of preparation. It is evident from SEM that drug, polymer, and excipients particles are merged or clustered together, as shown in Figure 4 [75]. Thus, it is justified that the drug and excipients are blended prudently [76].

### 3.11. Percentage Moisture Content

In the case of ELEHBR film, values of moisture content range from 4.25 ± 0.15% to 9.33 ± 0.25% for formulation F13 and F3, respectively. The presence of plasticizer and surfactant reduces the surface tension and relaxes the polymer chains, so it could be the cause of more entrapment of water molecules inside the meshwork of the polymer [77].

### 3.12. Disintegration Time (DT) and Total Dissolving Time (TDT)

DT for film ranges from 7 ± 1 s for formulation F12 to a maximum of 13 ± 1 s for formulation F4. DT has a direct relation with polymer concentration. As the polymer concentration increases, the stiffness of films increases and so DT increases [78]. It was evident by using polynomial Equation 6 and the 3 D plot, that both have a cumulative impact on film DT. The ANOVA for DT showed a value of R^2^ of 0.9082 and an adjusted R^2^ of 0.8163. The *p*-value was <0.05 (0.0125).

Polynomial Equation (7) describes that the TDT and polymer have a constructive impact on TDT. A negative sign for plasticizers represents a decrease in TDT [79]. TDT ranges from 40.5 ± 1 for F4 to a minimum of 22 ± 1 for F7. The ANOVA for TDT shows a value of R^2^ of 0.8566 and an adjusted R^2^ of 0.7113. The *p*-value was < 0.05 (0.0366).

DT = β_0_ + 9.63 + 1.72 X_1_ − 0.036 X_2_ – 0.32 X_1_X_2_ + 0.16 X_1_^2^ + 0.66 X_2_^2^ (6)

TDT = β_0_ + 25 + 4.42 X_1_ − 3.12 X_2_ − 0.63 X_1_X_2_ + 3.16 X_1_^2^ − 0.34 X_2_^2^ (7)

### 3.13. Mechanical Strength

#### 3.13.1. TS

**Figure 5:**
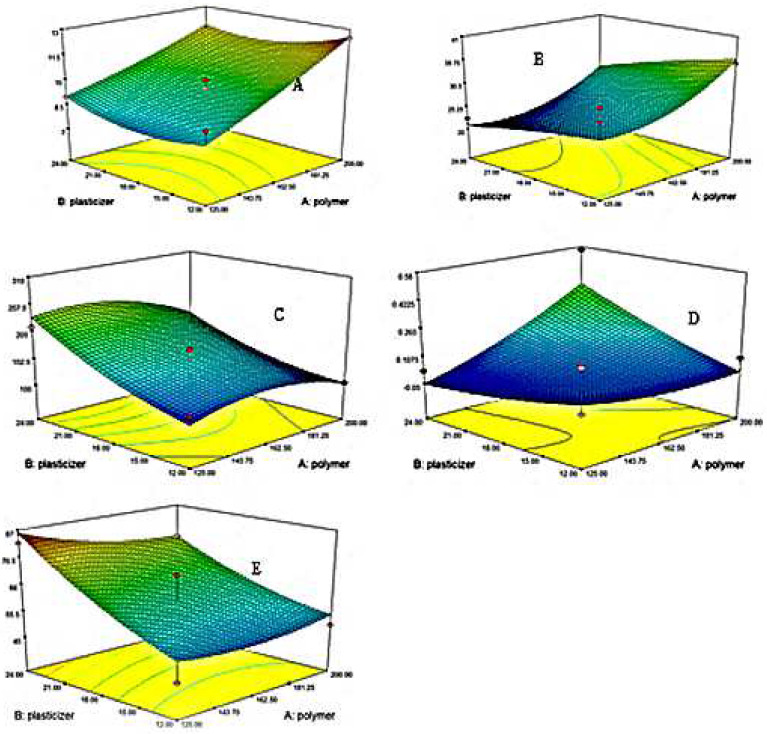
3D Contour plots represents (**A**) DT (**B**) TDT (**C**) FE (**D**) TS (**E**)%EB.

TS is affected by the concentration of plasticizers. Polynomial Equation (8) and 3D graph Figure 5 clearly showed that the higher the concentration of plasticizer, the lower the TS [80,81]. The incorporation of plasticizer reduces the inside chain entanglement of the polymer and ultimately increases the flexibility of the developed film [82]. The higher the concentrations of HPMC E5, the higher the TS [83,84]. HPMC E5 will possibly enhance the TS of the films [80].

TS = β_0_+ 0.050 + 0.28 X_1_ − 4.59×10^−0.005^ X_2_ + 0.036 X_1_X_2_ + 0.19 X_1_^2^ + 0.017 X_2_^2^ (8)

ANOVA for TS showed a value of R^2^ of 0.9656 and an adjusted R^2^ of 0.9313. The *p* was <0.05 (0.0011).

#### 3.13.2. % EB

From polynomial Equation (9) and 3D contour plot Figure 5, it was concluded that Tween 80 and glycerol have a synergistic impact on % EB of formulated films [85].

EB = β_0_+ 60.93 − 2.50 X_1_ + 13.28 X_2_ − 3.25 X_1_X_2_ − 2.97 X_1_^2^ + 2.97 X_2_^2^ (9)

By utilizing ANOVA for%, EB, the value of R^2^ was 0.8360, and adjusted R^2^ was 0.6721. The *p*-value was less than 0.05 (0.0491). As the% EB value increases, the films become more elastic, and a low value indicates tough films. So, an optimized elastic film must be ensured to be safe and effective during production, transportation, and administration.

#### 3.13.3. Strain

When a force is applied to films, it produces an elongation in them under the influence of force. So, strain defines the flexibility of established films [86]. It was evident from the polynomial equation 10 that the amount of plasticizer increases the system’s flexibility and polymer decreases it.

Strain = β_0_+ 39 − 2.47 X_1_ + 5.87 X_2_– 2.25 X_1_X_2_ + 2.09 X_1_^2^ + 0.97 X_2_^2^ (10)

Using ANOVA for strain, the value of R^2^ was 0.8485, and the adjusted R^2^ was 0.6970. The analysis was significant as the *p*-value was less than 0.05 (0.0409).

#### 3.13.4. FE

To confirm the flexible nature of films, they are folded at the same point approximately 200 to 300 times. If the formulated films have an FE value of more than 200, they are considered flexible [46]. In the case of EHBR films folding values ranged from 305 ± 1 time for formulation F8 to 105 ± 1 time for formulation F9. By engaging polynomial Equation (11) and 3 D contour plot Figure 5, it was obvious that the negative values of HPMC (X_1_) impart a negative effect and reduce the FE. Meanwhile, the positive values of X_2_ showed that plasticizer increases the flexibility of formulated films.

FE = β_0_+ 169 − 14.16 X_1_ + 50.46 X_2_ − 11.25 X_1_X_2_ − 26.69 X_1_^2^ + 15.81 X_2_^2^ (11)

Using ANOVA for FE, the value of R^2^ was 0.8990, and the adjusted R^2^ was 0.7980. The analysis was significant as the value of *p* was less than 0.05 (0.0157).

### 3.14. In Vitro Drug Release

**Figure 6:**
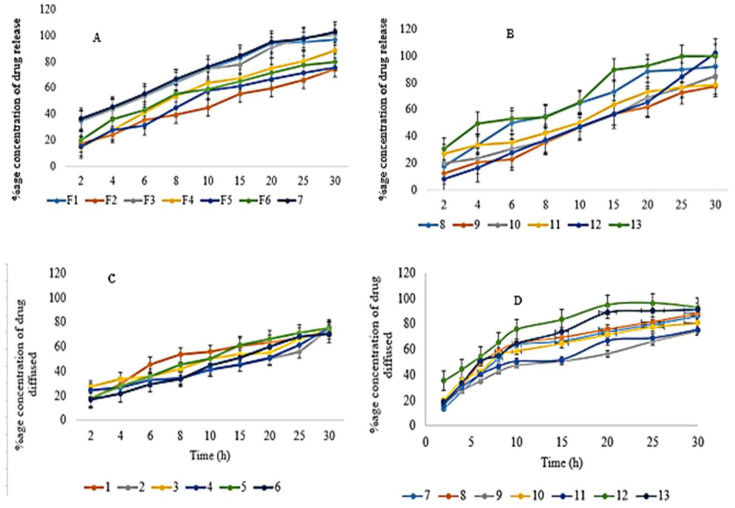
Represents (**A**) In vitro drug release for EHBR formulations from 1–7 (**B**) In vitro drug release for EHBR formulations from 8–13 (**C**) DD for EHBR formulations from 1–6 (**D**) DD for EHBR formulations from 7–13.

Formulated buccal films were developed for immediate drug release studies, so a 30-min study was conducted. A drug release of a maximum of 102.61 ± 1.13% for F7 and the lowest release of 74.5 ± 1.15% for F2 was observed, as shown in Figure 6. Using polynomial Equation (12), the X1 variable has a negative value, indicating that the drug release would decrease if the polymer concentration increased [79]. The possible justification was that the increased polymer concentration increases the polymer meshwork, and the drug’s release decreases [87]. HPMC E5 is a hydrophilic polymer used as a film-forming agent. Its increased concentration makes a thick and more concentrated gel around the drug molecules, which retards the release of drug components. Using ANOVA for drug release, the value of R^2^ was 0.9013, and the adjusted R^2^ was 0.8025. The analysis was significant as the value of *p* was less than 0.05 (0.0149).

Drug release = β_0_+ 76.97 − 3.15 X_1_ + 3.93 X_2_ + 3.00 X_1_X_2_ + 9.58 X_1_^2^ + 9.33 X_2_^2^ (12)

### 3.15. Drug Diffusion (DD)

Glycerol was used as a plasticizer in the formulation of films. It provides the films with an elastic and plastic nature. As these plasticizers are hygroscopic, they drag water molecules from their surroundings and enhance the solubility and permeability of the formulated films; more water in the surroundings means more uptake and more drug disintegration [88].

Tween 80, which was used as a surfactant, also improves drug permeability. It encompasses the skin membrane parameters to enhance permeability, for instance, causing conjugation of molecules at the skin site. In addition, they decrease the stratum corneum properties as a skin barrier, and they modify the skin by loosening the lipid layer, increasing permeability [89].

A DD of 92.87 ± 0.87% for F12 and the lowest release of 70.3 ± 1.13% for F6 was observed, as shown in Figure 6. Using ANOVA, the drug diffusion value of R^2^ was 0.8468, and the adjusted R^2^ was 0.6936. The analysis was significant as the value of *p* was less than 0.05 (0.0420). The polynomial Equation (13) clearly shows the negative impact of polymer on drug diffusion.

DD = β_0_+ 74.65 − 5.88 X_1_ +0.43 X_2_– 0.62 X_1_X_2_ + 7.38 X_1_^2^ + 5.57 X_2_^2^ (13)

### 3.16. Kinetic Modeling

Drug release profiling of all formulated films was analyzed for first order, zero order, Korsmeyer model, Hixson Crowell model, Higuchi model, and the value of n, as shown in Table 3. Formulations F4 and F8 follow first-order kinetics, while the remaining formulations follow the Korsmeyer Peppas model. The n value ranges between 0.378 to 0.807. Suppose the value on n is less than 0.5. In that case, it indicates the Fickian model and drug release followed the diffusion mechanism, which states that the solvent transportation and diffusion rate was far larger than the development of polymeric chain leisure. Moreover, if the value of n is greater than 0.5 but less than 0.89, it follows the non-Fickian or anomalous type of transport mechanism. Both swelling and diffusion mechanism was favored for drug release.

### 3.17. Model-Independent Approach

As per the recommendations of the Food and Drug Administration (FDA), if the similarity factor of f2 falls between 50 to 100, we declare the two formulations as similar [90]. Both f1 and f2 have an important function when we compare dissolution profiling. The f2 value should be nearer to 100 when the formulation is identical to the reference formulation. The value of f2 ranges from 0 to 100 and a formulation is considered similar if the value falls between 50 to 100 [91]. Shah et al. conducted a study and reported that if the calculated value of f2 would be between 50 to 65 of the dissolution profile, the formulations were similar with only a difference of 10 to 5% respectively between reference and test formulation [92]. In the case of drug release, when computed for formulations of EHBR, there appeared a similarity of up to 75% with formulation F5 and a maximum dissimilarity of 50% with formulation F7. In a study conducted by M. C. Gohe et al., they described the similarity of their prepared formulation with the reference up to 75% [92]. While comparing the optimized formulation with test formulations in the case of DD, the results depict that formulation F6 was 68% similar, and F12 was 53% dissimilar.

### 3.18. Numerical Optimization

The formulation was optimized based on the lowest DT time; the lowest DT time indicated rapid drug release. In addition, a lower TDT time will result in a high drug release of DD. In the optimized formulation, the quantity of polymer plasticizer was so adjusted that DT and TDT would be lowered. Results of the optimized formulation show a DT of 10.3 ± 2.30, while TDT was 24 ± 1.2 s. Cumulative drug release was 101 ± 1.5%, and DD was 79.03 ± 0.65%.

### 3.19. Ex Vivo Permeation

Ex vivo permeation was conducted on optimized formulations, using fresh goat buccal mucosa. During the permeation studies of 30 min, 80.9% drug was found to be permeated across the buccal mucosa describing the prompt permeation of the drug and satisfying the objectives of the study.

### 3.20. In Vivo Pharmacokinetics

**Figure 7:**
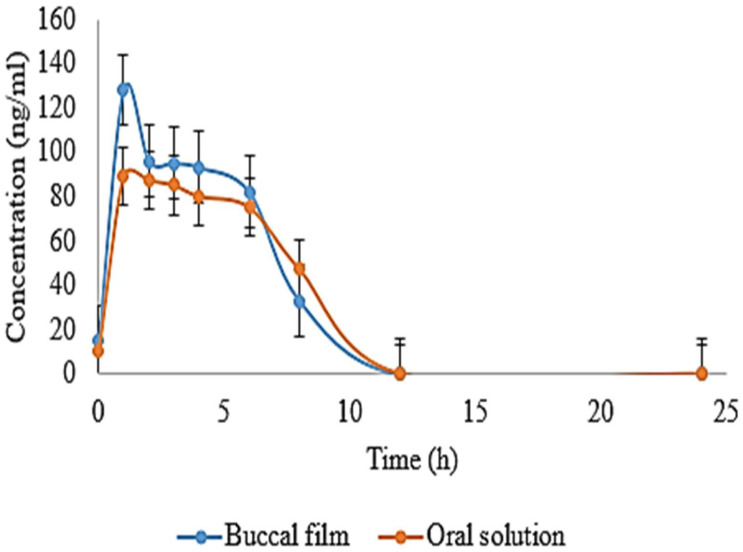
Represents mean drug concentration for a comparison between oral solution and film.

Pure drug solution of EHBR was evaluated with formulated EHBR film. HPLC method was previously validated for accuracy, precession, sensitivity, and assay for both EHBR in film and solution form. Figure 7 represents the difference between an oral solution and administered film. A high release of the drug was observed in the case of buccal film. A remarkable drug was detected at the end of the 8th hour. Pharmacokinetic parameters, such as t_1/2_, T_max_, C_max_, AUC 0-t, and MRT of oral solution and film, were studied and compiled in Table 4. The pharmacokinetic parameters suggest a relatively higher drug release profile with buccal film than the oral solution. The aim to formulate a buccal film providing immediate release appeared fulfilled.

Furthermore, a t-test was applied to the results compiled in Table 5. The *t*-test involves the null hypothesis, which states that there appeared no difference in the mean of the compared samples. *p*-value at a value of 0.05 was also calculated, which is linked with the null hypothesis by considering the tails of the test at both ends. When we compared two samples using a *t*-test, we assumed that there was no difference between the means of both samples or that the mean of one was higher than the other. The possible reason for adopting the two-tailed t-test was that it could reject or accept the null hypothesis [93]. When we compared the parameters, such as t_1/2_, C_max_, and AUC, it was observed that the result of both parameters was significant as the value of *p* < 0.05.

### 3.21. Stability Studies

Optimized formulations were kept at 40 °C and 75% ± 5% RH and 30 °C and 65% ± 5% for 3 months. Parameters that include DT, TDT, drug content, color, in vitro drug release, and DD studies were conducted in the 1st and 3rd months. Results are represented in Table 6. There appeared no significant changes, which suggests that our formulation is suitable for a long period of storage.

### 3.22. Histopathological Slides

**Figure 8:**
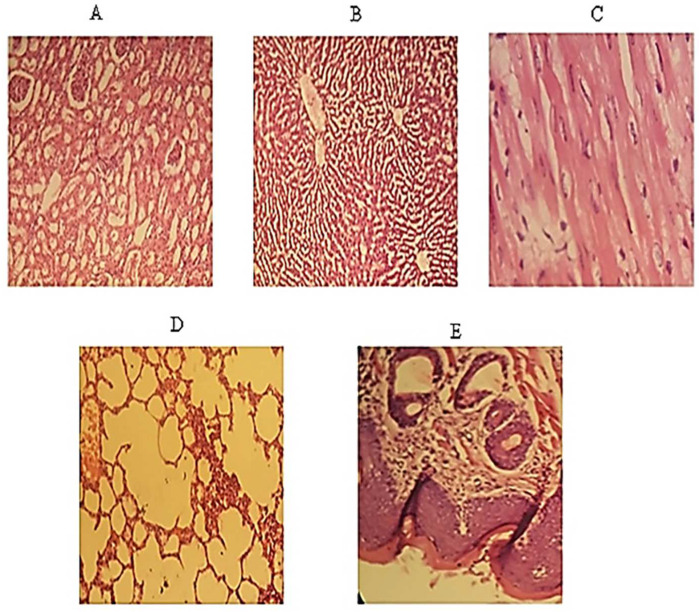
Represents histopathological images of (**A**) Kidney (**B**) Liver (**C**) Heart (**D**) Lung (**E**) Buccal mucosa.

The histopathological images of the kidney, liver, heart, lung, and buccal mucosa were prepared, as shown in Figure 8. The tissues of the kidneys were normal, and no sign of localized or general inflammation in the tubular or ductal cells was observed. In the case of the liver, well-defined hepatocytes were observed, and no sign of inflammation or cytoplasmic ballooning was investigated. However, fat deposition was observed in the sinusoids, but overall tissue health was good. Likewise, no sign of infection, necrosis, or cardiomegaly was noted in cardiomyocytes. Normal lung tissue with expanded alveoli with appropriate thickness was observed with no fluid deposition, granuloma, or emphysema symptoms. Overall, tissue health was good. The buccal mucosa was normal, with no eruption at the surface. All layers of the cells were in a good state. There was no sign of hyperactivity or accumulation of immune cells indicating inflammation.

## 4. Conclusions

All of the results mentioned above declared the suitability for the formulation of EHBR in the form of buccal film. The suggested or optimized formulation has successfully achieved immediate release drug delivery, rapidly improving migraine conditions and enhancing patient compliance.

## Figures and Tables

**Table 1:** Factors studied by RSM for preparation of EHBR-loaded films.

Independent Factors		Levels			
	−α	−1	0	+1	+α
Polymer	109.47	125	162.5	200	215.53
Plasticizer	9.5	12	18	24	26.49
Surfactant		Constant 5% of polymer concentration			

**Table 2:** Calculated formulation designs for preparation of EHBR films.

Formulation No	Polymer (HPMC E5) (mL)	Plasticizer (Glycerol) (mL)	(Surfactant Tween 80) (mL)
1	3.125	0.6	0.31
2	4.06	0.58	0.4
3	5	0.96	0.5
4	5.4	0.78	0.54
5	4.06	0.58	0.4
6	4.06	0.58	0.4
7	4.06	0.86	0.4
8	5	0.48	0.5
9	4.06	0.58	0.4
10	4.06	0.31	0.4
11	4.06	0.58	0.4
12	2.74	0.4	0.27
13	3.125	0.3	0.31

**Table 3:** Represents the R^2^ and value of all formulated films.

Formulation No	Zero Order	First Order	Highuci Model	Hixson Crowell Model	Korsmeyer Peppas Model	n	Best Fit Model
1	0.2783	0.9648	0.8918	0.9178	0.9650	0.379	Korsmeyer Peppas
2	0.5023	0.8981	0.9892	0.8130	0.9892	0.497	Korsmeyer Peppas
3	0.1842	0.9469	0.9182	0.8919	0.9789	0.388	Korsmeyer Peppas
4	0.4648	0.9604	0.9381	0.8983	0.9381	0.495	First-order
5	0.4120	0.9010	0.9342	0.8076	0.9351	0.483	Korsmeyer Peppas
6	0.0521	0.8387	0.9109	0.6768	0.9527	0.402	Korsmeyer Peppas
7	0.2820	0.9605	0.9014	0.9223	0.9761	0.378	Korsmeyer Peppas
8	0.3889	0.9878	0.9482	0.9532	0.9501	0.475	First-order
9	0.8158	0.9834	0.9390	0.9622	0.9728	0.637	Korsmeyer Peppas
10	0.7848	0.9768	0.9704	0.9577	0.9924	0.604	Korsmeyer Peppas
11	0.2663	0.8704	0.9629	0.7677	0.9721	0.449	Korsmeyer Peppas
12	0.9571	0.9405	0.8778	0.9661	0.9846	0.807	Korsmeyer Peppas
13	0.0822	0.9368	0.9309	0.9149	0.9564	0.420	Korsmeyer Peppas

**Table 4:** Pharmacokinetic parameters were evaluated and are presented.

Pharmacokinetic Parameters	Film (Mean ± SD)	Oral Solution (Mean ± SD)
t_1/2_ (h)	4.54 ± 0.18	5.20 ± 0.21
T_max_ (h)	1 ± 0.04	1 ± 0.39
C_max_ (ng/ml)	128 ± 0.87	89.5 ± 0.26
AUC 0-t (ng/mL×h)	663 ± 1.02	644.75 ± 1.15
MRT (h)	6.15 ± 0.24	7.92 ± 0.15

(mean ± standard deviation (SD) when n = 6).

**Table 5:** Possible outcomes after applying t-test.

*t*-Test	Mean Diff.	*p* Value	R^2^
C_max_			
Film vs. oral solution	38.50	<0.0001	0.9998
t_1/2_			
Film vs. oral solution	0.6600	0.0007	0.9986
AUC			
Film vs. oral solution	18.25	<0.0001	1.000

**Table 6:** Stability profiling of films.

Stability Profiling of Films at 40 °C and 75% RH after 1st Month					
DT	TDT	Drug Content	In Vitro Drug Release	DD Studies	Color
10.1 ± 1.30	24 ± 1.2	99.75%	99 ± 1.25	79.1 ± 1.0	No change
Stability profiling of films at 40 °C and 75% RH after 3rd month					
9.1 ± 0.30	22 ± 1.5	97.85%	98.35 ± 0.5	78.1 ± 1.5	No change
Stability profiling of Films at 30 °C and 65% RH at the end of 1st month					
10.1 ± 1.30	23 ± 1.5	101.35%	99.3 ± 1.15	79.13 ± 1.05	No change
Stability profiling of Films at 30 °C and 65% RH after 3rd month					
10.0 ±0.75	22.45 ± 0.5	99.25%	98.25 ± 0.24	79.3 ± 0.65	No change

## Data Availability

Not applicable.
